# Hemogram-derived ratios as prognostic markers of ICU admission in COVID-19

**DOI:** 10.1186/s12873-021-00480-w

**Published:** 2021-07-27

**Authors:** Sara Velazquez, Rodrigo Madurga, José María Castellano, Jesús Rodriguez-Pascual, Santiago Ruiz de Aguiar Diaz Obregon, Sara Jimeno, Juan Ignacio Montero, Paula Sol Ventura Wichner, Alejandro López-Escobar

**Affiliations:** 1 Anaesthesia Department, Hospital Universitario HM Sanchinarro, Madrid, Spain; 2 Anaesthesia Department, Hospital Universitario Santa Cristina, Madrid, Spain; 3 Fundación de Investigación HM Hospitales, Madrid, Spain; 4 Facultad de Medicina, Universidad CEU San Pablo, Madrid, Spain; 5 Faculty of Experimental Sciences, Universidad Francisco de Vitoria, Madrid, Spain; 6 Cardiology Department, Hospital Universitario HM Montepríncipe, Madrid, Spain; 7 Centro Nacional de Investigaciones Cardiovasculares, Instituto de Salud Carlos III, Madrid, Spain; 8 Oncology Department, HM-CIOCC, Hospital Universitario HM Puerta del Sur, Móstoles, Madrid, Spain; 9 Medical Management, Grupo HM Hospitales, Madrid, Spain; 10 Pediatrics Department, Hospital Universitario HM Puerta del Sur, Móstoles, Madrid, Spain; 11 Unidad de Investigación Clínica, Fundación Vithas. Grupo Vithas, Madrid, Spain; 12 Intensive Care Unit, Hospital Universitario HM Puerta del Sur, Móstoles, Madrid, Spain; 13 Pediatrics Department, Hospital Universitario HM Nens, Barcelona, Spain; 14 Fundació Institut d’Investigació en Ciències de la Salut Germans Trias i Pujol, Barcelona, Spain; 15 Pediatrics Department, Hospital Vithas Madrid La Milagrosa, Madrid, Spain

**Keywords:** COVID-19, Hemogram, Hemogram-derived ratio, Neutrophil-to-platelet ratio, Neutrophil-to-lymphocyte ratio, ICU admission

## Abstract

**Background:**

The vast impact of COVID-19 call for the identification of clinical parameter that can help predict a torpid evolution. Among these, endothelial injury has been proposed as one of the main pathophysiological mechanisms underlying the disease, promoting a hyperinflammatory and prothrombotic state leading to worse clinical outcomes. Leukocytes and platelets play a key role in inflammation and thrombogenesis, hence the objective of the current study was to study whether neutrophil-to-lymphocyte ratio (NLR), platelets-to-lymphocyte ratio (PLR), the systemic immune-inflammation index (SII) as well as the new parameter neutrophil-to-platelet ratio (NPR), could help identify patients who at risk of admission at Intensive Care Units.

**Methods:**

A retrospective observational study was performed at HM Hospitales including electronic health records from 2245 patients admitted due to COVID-19 from March 1 to June 10, 2020. Patients were divided into two groups, admitted at ICU or not.

**Results:**

Patients who were admitted at the ICU had significantly higher values in all hemogram-derived ratios at the moment of hospital admission compared to those who did not need ICU admission. Specifically, we found significant differences in NLR (6.9 [4–11.7] vs 4.1 [2.6–7.6], *p* <  0.0001), PLR (2 [1.4–3.3] vs 1.9 [1.3–2.9], *p* = 0.023), NPR (3 [2.1–4.2] vs 2.3 [1.6–3.2], *p <*  0.0001) and SII (13 [6.5–25.7] vs 9 [4.9–17.5], *p <*  0.0001) compared to those who did not require ICU admission. After multivariable logistic regression models, NPR was the hemogram-derived ratio with the highest predictive value of ICU admission, (OR 1.11 (95% CI: 0.98–1.22, *p* = 0.055).

**Conclusions:**

Simple, hemogram-derived ratios obtained from early hemogram at hospital admission, especially the novelty NPR, have shown to be useful predictors of risk of ICU admission in patients hospitalized due to COVID-19.

**Supplementary Information:**

The online version contains supplementary material available at 10.1186/s12873-021-00480-w.

## Introduction

In December 2019, the first cases of pneumonia of an unknown cause emerged in the city of Wuhan, China. On January 7th a new coronavirus [1] was identified as the cause of what was named by the World Health Organization (WHO) as SARS-CoV-2 [2]. The syndrome developed after infection with this new virus is known as coronavirus disease (COVID-19) [3–5] and on March 11^,^ 2020, the WHO declared the outbreak a pandemic [6].

Approximately 80% of infected people have mild to moderate symptoms, while the remaining 20% present a more severe clinical course. Intensive Care Unit (ICU) admission due to COVID-19 due to an acute hypoxemic respiratory failure ranges from 5 to 32% [7–9]. The cause of respiratory failure is the development of Acute Respiratory Distress Syndrome (ARDS), and it presents as a diffuse pulmonary inflammatory injury that leads to an increase in pulmonary vascular permeability which in turn severely impairs adequate gas exchange [10]. Severe COVID-19 has been shown to occur in patients with endothelial injury which may activate, through a cytokine release, a hyperinflammatory and procoagulant state. White blood cells, neutrophils, lymphocytes and monocytes, are directly involved in this systemic inflammatory response while platelets are the main mediators of hemostasis.

It has been previously described that severe COVID cases share common analytical abnormalities including increased white blood cells and neutrophils count as well as low lymphocyte counts [11]. Hemogram-derived ratios such as neutrophil-to-lymphocyte ratio (NLR) and platelet-to-lymphocyte ratio (PLR) have been proposed to assess the extension of the systemic inflammation in this context. NLR has been shown to correlate with worse outcome in patients with SARS-Cov-2 infection [12–14]. PLR is a not only a marker of acute inflammatory and prothrombotic states but it has been shown to reflect the degree of cytokine release, which might prove useful as a prognostic marker in severe COVID-19 [15].

Systemic immune-inflammation-index (SII), has been used as a prognostic indicator in the follow-up of sepsis [16] and in cancer patients [17, 18].

In this study we propose to include a new hemogram derived ratio, the neutrophil-to-platelet ratio (NPR) which incorporates neutrophil count, which are implicated in the inflammatory response to infection and also involved in the thrombotic mechanism, and the platelet count which play a pivotal role in thrombosis [19].

Risk factors associated with severe course of COVID-19 have been described, but there are few that refer specifically to the risk of admission to the ICU [14].

Previous data published by our group suggest role for these hemogram-derived ratios in the early identification of severe COVID-19 cases with a high risk of in-hospital mortality [12, 14]. Building on these previous results, the main objective of the current work is to further elucidate the role of hemogram-derived ratios as prognostic markers of severe COVID-19 defined as the need for ICU admission. Secondary outcomes include the identification of other variables related to the risk of ICU admission.

## Materials and methods

COVID-19 patients that required hospitalization at any of the tertiary hospitals of the Grupo HM Hospitales between March 1 and June 10, 2020, were retrospectively included in the study. Demographic data, comorbidities, epidemiological characteristics and laboratory findings from each patient were collected from the electronic health report system at hospital admission. Last follow up was on June 24, 2020. A total of 2543 COVID-19 patients were admitted during the study period.

The study protocol was approved by the HM Hospitales ethics committee on March 25, 2020 (approval number 20.03.1573-GHM).

All patients were assessed at the Emergency Department where blood sample was drawn. Laboratory assessments consisted of complete blood count (including white blood cell count, leukocyte subtypes, hemoglobin count and platelet count), biochemical parameters and blood coagulation tests (including D-dimer, prothrombin time and activated partial prothrombin time).

Counts of neutrophils (× 10^9^ cells/L), lymphocytes (× 10^9^ cells/L) and platelets (× 10^11^ cells/L) were used to define the hemogram-derived ratios, NLR is the ratio between neutrophils and lymphocytes, PLR is the ratio between platelets and lymphocytes, NPR is the ratio between neutrophils and platelets and, finally, SII is defined as neutrophils multiplied by platelets and divided by lymphocytes.

Consecutive laboratory tests obtained the first 3 days after admission allowed to measure the rate of change of these ratios. The rate of change compares the evolution of NLR, PLR, NPR and SII with the value obtained at admission and was defined as the slope of the linear fit of the relative rates versus time from hospital entry in days. It is considered a positive change if the value of the ratio is increased more than 10% per day, and negative if the value decreased at least 10% per day. In those cases were the change was between − 10 and 10% per day the rate was classified as null.

Summary statistics were made for the entire cohort and the patients who needed to be admitted to the ICU and those who were not were grouped together. Continuous variables were summarized as median (interquartile range) and categorical variables as absolute frequency (relative frequency, %).

Differences between groups were evaluated applying Mann-Whitney U test for quantitative variables and Χ^2^ test or Fisher’s exact test for categorical variables. Spearman’s rho test was used to evaluate correlation between continuous variables. Correlation plots were constructed using the R package *GGally*.

Variables that showed differences between both groups, admitted vs. not admitted to ICU, with *p* value < 0.2 were selected for univariable logistic regression.

To implement the bivariable logistic regression models the ratios were conflated to other variables. Those parameters that added to the model, modifying the value of the ratios by at least 10%, were included in the multivariable adjusted models. Model A included age, heart rate, temperature > 38 °C, systolic and diastolic blood pressure, NLR rate of change > 10% per day, AST, D-Dimer and glucose. Models B-D included the previous model and additionaly, oxygen saturation (> 94, 90–94 or < 90%), lactate dehydrogenase (LDH) or C-Reactive-Protein (CRP), respectively.

**Fig. 1:**
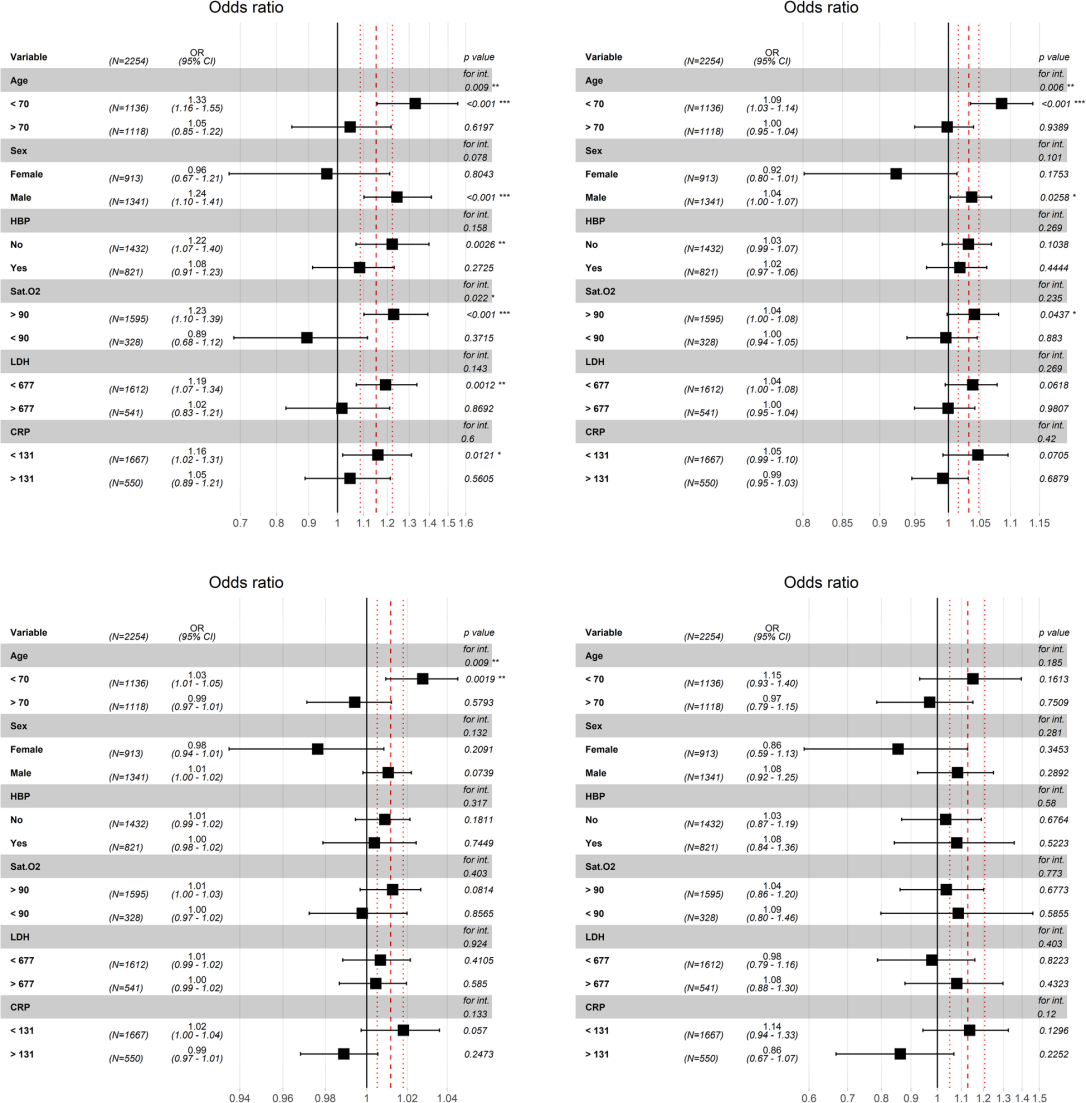
A. Interactions and stratified analyses for NPR (neutrophil-platelets ratio) adjusted to model A (Table 5) and conducted for age (< 70 and > 70 years), sex, High blood pressure (HBP), oxygen saturation (< 90 and > 90%) (SaO_2_), and lactate dehydrogenase (LDH) and C-reactive protein (CRP) both categorized through their respective median values

Interaction and stratified analyses were performed for each hemogram-derived ratio adjusted to model A and conducted for age (< 70 and > 70 years), sex, hypertension, diabetes mellitus, oxygen saturation (< 90 and > 90%) and LDH and CRP both categorized through their respective median values (Figs. 1A, S1B, S1C, S1D).

Statistical inference was performed using two-tailed test and with type I error rate of 0.05. All statistical analyses were done using R (version 4.0.0).

## Results

The clinical and laboratory data of 2254 patients admitted to Grupo HM Hospitales due to COVID-19 infection during the study period, were collected. Patients under 18 years old (*n* = 5), missing laboratory data within the first 24 h of admission (*n* = 258), or who died at hospital admission (*n* = 26) were excluded for the analysis. A total of 2254 (88.6%) were included in the final analysis as shown in the flow diagram in Fig. S2.

Clinical characteristics are summarized in Table 1 and laboratory findings in Tables 2 and 3. Median age was 69 [57–80] and 59.5% were men. At the time of admission, all patients were assessed at the Emergency Department and blood sample was drawn for analysis. All received standard approved treatment for COVID-19 according to current guidelines.

**Table 1:** Demographics and Clinical characteristics (% and median value (interquartile range))

	Total (***n*** = 2254)	ICU (***n*** = 185)	Non-ICU (***n*** = 2069)	***P*** value	Univariate analysis	
					OR (95% CI)	***P*** value
**Demographics characteristics**						
Age (years)	69 (57–80)	68 (61–74)	70 (57–81)	0.057	0.99 (0.98–1.00)	0.18
Male (%)	59.5%	74.6%	58.1%	< 0.0001	**2.11 (1.51–3.00)**	< 0.0001
**Comorbidities**						
HBP	36.4%	43.8%	35.8%	**0.032**	1.41 (1.04–1.91)	**0.026**
DM	18.1%	21.6%	17.7%	0.21	NA	NA
COPD	5.7%	4.3%	5.8%	0.52	NA	NA
CD	11.5%	11.9%	11.5%	0.95	NA	NA
**Clinical Characteristics**						
Temperature > 38 °C (%)	6.9%	14.1%	130 (6.3%)	< 0.0001	2.77 (1.72–4.34)	< 0.0001
Heart rate (bpm)	89 (78–101)	91 (84–101)	89 (78–101)	0.10	NA	NA
BP max (mm Hg)	131 (117–146)	130 (119–140)	131 (117–146)	0.36	NA	NA
BP min (mm Hg)	76 (67–84)	66–83)	76 (67–84)	0.56	NA	NA
Sa O2 (%) > 94%	50.4%	29.2%	52.2%	< 0.0001	NA	NA
Sa O2 (%) 90–94%	20.4%	21.6%	20.3%	< 0.0001	1.91 (1.24–2.91)	0.0029
Sa O2 (%) < 90%	14.6%	22.7%	13.8%	< 0.0001	2.94 (1.92–4.48)	< 0.0001
**In-hospital mortality**	14.7%	33%	13%	**< 0.0001**	NA	NA

ICU-admitted vs Non-ICU admitted.

*Abbreviations*: *ICU* Intensive Care Unit, *HBP* High blood pressure, *DM* Diabetes Mellitus, *COPD* Chronic obstructive pulmonary disease, *CD* cardiovascular disease, *BP* Blood pressure, *Sa O2* oxygen saturation

**Table 2:** Laboratory findings at admission. Median value (interquartile range)

	Total (n: 2254)	ICU (***n*** = 185)	Non-ICU (***n*** = 2069)	***p*** value	Univariate analysis	
					OR (95% CI)	***P*** value
**Laboratory findings**						
**White blood cells (10^9/L)**	6.6 (5.0–8.9)	7.2 (5.4–10.3)	6.6 (5.0–8.8)	**0.0034**	**1.04 (1.01–1.08)**^**a**^	**0.012**
**Red blood cells (10^12/L)**	4.7 (4.2–5.0)	4.7 (4.2–5.0)	4.7 (4.2–5.0)	0.85	NA	NA
**Neutrophils (10^9/L)**	4.8 (3.3–6.9)	5.7 (4.0–8.6)	4.7 (3.3–6.7)	**< 0.0001**	**1.07 (1.03–1.10)**^**a**^	**0.00014**
**Lymphocytes (10^9/L)**	1.1 (0.8–1.5)	0.9 (0.6–1.2)	1.1 (0.8–1.5)	**< 0.0001**	**0.59 (0.44–0.78)**^**a**^	**0.00036**
**Monocytes (10^9/L)**	0.5 (0.3–0.7)	0.4 (0.3–0.6)	0.5 (0.3–0.7)	**< 0.0001**	**0.27 (0.14–0.48)**^**a**^	**< 0.0001**
**Platelets (10^9/L)**	205 (159–266)	186 (150–240)	208 (161–268)	**0.0034**	1.00 (1.00–1.00)	0.072
**Hemoglobin (g/dL)**	13.8 (12.5–14.9)	13.9 (12.5–15.1)	13.8 (12.5–14.9)	0.37	NA	NA
**MCHC (g/dL)**	33.7 (32.8–34.5)	34.0 (33.3–34.8)	33.6 (32.7–34.4)	**< 0.0001**	**1.27 (1.14–1.42)**^**a**^	**< 0.0001**
**AST (U/L)**	31.6 (22.3–49.2)	43.1 (29.0–70.7)	31.0 (21.8–47.5)	**< 0.0001**	**1.01 (1.01–1.01)**^**a**^	**< 0.0001**
**ALT (U/L)**	25.5 (16.1–42.4)	32.0 (20.3–49.0)	25.0 (15.7–41.4)	**< 0.0001**	1.00 (1.00–1.00)	0.095
**Creatinine (mg/dL)**	0.9 (0.7–1.1)	1.0 (0.8–1.2)	0.9 (0.7–1.1)	**0.0042**	1.10 (0.88–1.31)	0.30
**LDH (U/L)**	521 (397–677)	675 (532–931)	510 (390–655)	**< 0.0001**	**1.00 (1.00–1.00)**^**a**^	**< 0.0001**
**C-reactive protein (mg/L)**	64 (24–131)	117 (59–225)	61 (22–122)	**< 0.0001**	**1.01 (1.00–1.01)**^**a**^	**< 0.0001**
**Urea (mg/dL)**	34.7 (26.0–49.4)	36.3 (27.8–50.6)	34.5 (26.0–49.3)	0.18	NA	NA
**Glucose (mg/dL)**	114 (100–137)	125 (111–150)	113 (100–135)	**< 0.0001**	**1.01 (1.00–1.01)**^**a**^	**< 0.0001**
**Partial thromboplastin time (s)**	32 (30–35)	32 (30–34)	32 (30–35)	0.18	NA	NA
**D-dimer (mg/L)**	1 (0–1)	1 (1–2)	1 (0–1)	**0.0032**	**1.03 (1.01–1.04)**^**a**^	**0.00030**
**Prothrombin time (s)**	13.3 (12.3–14.5)	13.4 (12.6–14.7)	13.2 (12.3–14.5)	0.24	NA	NA

^a^The variable is continuous, the OR is for each increment in a unit. ICU-admitted vs Non-ICU admitted.

*Abbreviations*: *ICU* Intensive Care Unit, *MCHC* mean corpuscular hemoglobin concentration, *MCV* Mean corpuscular volume, *MPV* Mean platelet volume, *NLR* neutrophil-lymphocyte ratio, *PLR* platelet-lymphocyte ratio, *NPR* neutrophil-platelets ratio, *SII* systemic immune-inflammation index, *AST* Aspartate aminotransferase, *ALT* lactate aminotransferase, *LDH* lactate dehydrogenase

**Table 3:** Hemogram hemogram-derived ratios findings. Median value. (interquartile range)

	Total (***n*** = 2254)	ICU (***n*** = 185)	Non-ICU (***n*** = 2069)	***p*** value	Univariate analysis	
					OR (95% CI)	***P*** value
**Hemogram hemogram-derived ratio at admission**						
NLR	4.3 (2.7–8.0)	6.9 (4.0–11.7)	4.1 (2.6–7.6)	**< 0.0001**	**1.03 (1.02–1.05)**^**a**^	**< 0.0001**
PLR	1.9 (1.3–2.9)	2.0 (1.4–3.3)	1.9 (1.3–2.9)	**0.023**	**1.13 (1.05–1.21)**^**a**^	**0.00057**
NPR	2.3 (1.6–3.3)	3.0 (2.1–4.2)	2.3 (1.6–3.2)	**< 0.0001**	**1.15 (1.09–1.22)**^**a**^	**< 0.0001**
SII	9.2 (5.0–18.2)	13.0 (6.5–25.7)	9.0 (4.9–17.5)	**< 0.0001**	**1.01 (1.01–1.02)**^**a**^	**0.00028**
**Positive rate of change (> 10% · day^-1)**						
NLR	20.9%	37.8%	19.4%	**< 0.0001**	**1.87 (1.33–2.65)**	**0.00036**
PLR	27.2%	35.7%	26.5%	0.33	NA	NA
NPR	42.7%	60%	41.1%	**0.0029**	**1.74 (1.26–2.42)**	**0.00087**
SII	30%	44.9%	28.7%	**0.003**	**1.55 (1.11–2.19)**	**0.011**

^a^The variable is continuous, the OR is for each increment in a unit. ICU-admitted vs Non-ICU admitted. The rate of change of the different inflammation ratios was obtained with up to four consecutive blood cells measurements since hospital entry. The rate of change was defined as the slope of the linear fit of the relative rates versus time from hospital entry in days. A rate of change higher than 10% per day was considered as positive.

*Abbreviations*: *ICU* Intensive Care Unit, *NLR* neutrophil-lymphocyte ratio, *PLR* platelet-lymphocyte ratio, *NPR* neutrophil-platelets ratio, *SII* systemic immune-inflammation index

Infection by SARS-CoV-2 was confirmed by PCR in 2114 (93.8%) patients. The remaining 140 patients included presented clinical (severe acute respiratory infection) and/or radiological signs compatible with COVID-19, as per protocol.

One hundred and eighty five patients (8.29%) experienced severe acute respiratory failure and were admitted to the ICU. Three hundred and thirty one patients (14.7%) passed away, 61 (33%) from the ICU group and 270 (13%) from non-ICU group (*p <*  0.0001).

At the time of hospital admission, clinical differences were observed between patients who were admitted to ICU and those who were not admitted, including sex (74.6% vs 58.1% males, odds ratio [OR]: 2.11; 95% CI: 1.51–3; *p <*  0.0001), temperature above 38 °C (14.1% vs 6.3%, odds ratio [OR]: 2.77; 95% CI: 1.72–4.34; *p <*  0.0001) and oxygen saturation (SaO2 < 90 22.7% vs 13.8%, odds ratio [OR]: 2.94; 95% CI: 1.92–4.48; *p <*  0.0001). Patients admitted to the ICU were more often hypertensive than those not requiring ICU admission (43.8% vs 35.8%, odds ratio [OR]: 1.41; 95% CI: 1.04–1.91; *p* = 0.026) (Table 1).

The differences in hemogram derived rations between ICU admitted patients and those not requiring ICU admission are shown in Tables 2 and 3. Patients requiring ICU admission had significantly higher hemogram-derived ratios at the time of hospital admission compared to patients not requiring ICU admission including NLR (6.9 [4–11.7] vs 4.1 [2.6–7.6], *p* <  0.0001), PLR (2 [1.4–3.3] vs 1.9 [1.3–2.9], *p* = 0.023), NPR (3 [2.1–4.2] vs 2.3 [1.6–3.2], *p <*  0.0001) and SII (13 [6.5–25.7] vs 9 [4.9–17.5], *p <*  0.0001) than those who were not admitted in the ICU (Table 3).

Independent mortality prediction ability was shown for each hemogram-derived ratio (ROC curves are shown in Fig. S3 and optimal cut-off values are shown in Table 4).

**Table 4:** Optimal cut-off values for the different immunoinflammatory ratios with their sensitivities and specificities and their corresponding 95% confidence interval

Variable	Cut-off	Sensitivity	Specificity
NLR	4.93	0.68 (0.49–0.80)	0.58 (0.47–0.74)
PLR	2.50	0.47 (0.19–0.85)	0.66 (0.27–0.89)
NPR	2.44	0.68 (0.54–0.77)	0.58 (0.54–0.71)
SII	12.26	0.55 (0.32–0.87)	0.64 (0.30–0.84)

*Abbreviations*: *NLR* neutrophil-lymphocyte ratio, *PLR* platelet-lymphocyte ratio, *NPR* neutrophil-platelets ratio, *SII* systemic immune-inflammation index

Patients requiring ICU admission showed a significantly higher rate of ascent in the velocity of NLR (37.8% vs 19.4% odds ratio [OR]: 1.87; 95% CI: 1.33–2.65, *p <*  0.0001), NPR (60% vs 41.1% odds ratio [OR]: 1.74; 95% CI: 1.26–2.42, *p* = 0.0029) and SII (44.9% vs 28.7% odds ratio [OR]: 1.55; 95% CI: 1.11–2.19, *p* = 0.0032), but not in the rate of PLR (35.7% vs 26.5%, *p* = 0.33) (Table 3).

The results of multivariable logistic regression models assessing the relation of the different hemogram-derived ratios and requiring ICU admission are shown in Table 5. Model A adjusted the hemogram-derived ratios OR for age, heart rate, temperature > 38 °C, systolic and diastolic blood pressure, NLR rate of change > 10% per day, AST, D-Dimer, and glucose. This adjustment made every ratio to lose their association with ICU admission except NPR, which obtained borderline significance in the most robust model (model D, *p* = 0.055) (Table 5).

**Table 5:** Multivariable adjusted models

Model		NLR	PLR	NPR	SII
**Unadjusted**	OR (95% CI)	1.03 (1.02–1.05)	1.13 (1.05–1.21)	1.15 (1.09–1.22)	1.01 (1.01–1.02)
	*p* value	**< 0.0001**	**0.00057**	**< 0.0001**	**0.00028**
**Model A**	OR (95% CI)	1.02 (0.993–1.05)	1.04 (0.903–1.17)	1.15 (1.05–1.25)	1.01 (0.995–1.02)
	*p* value	0.11	0.58	**0.0018**	0.19
**Model A + SaO2**	OR (95% CI)	1.02 (0.985–1.05)	1.03 (0.888–1.18)	1.14 (1.03–1.25)	1.01 (0.993–1.02)
	*p* value	0.24	0.65	**0.0044**	0.31
**Model B + LDH**	OR (95% CI)	1.02 (0.983–1.05)	1.03 (0.885–1.17)	1.14 (1.03–1.24)	1.01 (0.992–1.02)
	*p* value	0.3	0.68	**0.0061**	0.37
**Model C + CRP**	OR (95% CI)	0.998 (0.961–1.03)	0.979 (0.831–1.13)	1.11 (0.986–1.22)	0.996 (0.98–1.01)
	*p* value	0.93	0.78	0.055	0.64

Model A: Age, heart rate, temperature > 38 °C, systolic and diastolic blood pressure, NLR rate of change > 10% per day, AST, D-dimer and glucose. Model B: Model A + Oxygen Saturation (Sa O_2_). Model C: Model B + LDH. Model D: Model C + CRP.

*Abbreviations*: *NLR* neutrophil-lymphocyte ratio, *PLR* platelet-lymphocyte ratio, *NPR* neutrophil-platelets ratio, *SII* systemic immune-inflammation index, *BP* blood pressure, *LDH* lactate dehydrogenase, *CRP* C-reactive protein

Stratified analysis showed that increasing values of NPR significantly associates with the risk of ICU admission for age < 70 years (odds ratio [OR]: 1.33; 95% CI: 1.16–1.55, *p* < 0.001), sex male (odds ratio [OR]: 1.24; 95% CI: 1.10–1.41, *p <* 0.001), absence of hypertension (odds ratio [OR]: 1.22; 95% CI: 1.07–1.40, *p* = 0.0026), SaO2 > 90% (odds ratio [OR]: 1.23; 95% CI: 1.10–1.39, *p <* 0.001), LDH below median (< 677 U/L) (odds ratio [OR]: 1.19; 95% CI: 1.07–1.34, *p =* 0.001) and CPR below median (< 131 mg/L) (odds ratio [OR]: 1.16; 95% CI: 1.02–1.31, *p* = 0.012), showing statistical significant interaction with age (*p =* 0.009) and SaO_2_ (*p* = 0.022) (Fig. 1A).

Higher values of NLR were significantly associated with ICU entry only for strata with SaO_2_ > 90% (odds ratio [OR]: 1.04; 95% CI: 1.00–1.08, *p* = 0.043), male sex (odds ratio [OR]: 1.04; 95% CI: 1.01–1.07, *p* = 0.025) and age < 70 years (odds ratio [OR]: 1.09; 95% CI: 1.03–1.14, *p =* < 0.001), the latter showing significant interaction (*p* = 0.006) (Fig. S1B).

Higher values of SII were almost significantly associated with risk of ICU admission for patients with CRP lower than 131 mg/L (median value) (odds ratio [OR]: 1.02; 95% CI: 1.00–1.04, *p* = 0.057) and for patients younger than 70 (odds ratio [OR]: 1.03; 95% CI: 1.01–1.05, *p* = 0.0019), with significant interaction found with age (*p =* 0.009) (Fig. S1C).

PLR showed no significant association with ICU admission in the stratified analysis (Fig. S1D).

Testing the dependency of the variables used for stratification using Chi-squared test, we obtained that being older or younger than 70 years is related with sex (*p <* 0.0001), HBP (high blood pressure) (*p <* 0.0001), SaO_2_ < 90% (*p <* 0.0001), LDH < 677 U/L (*p* = 0.0004) and CRP < 131 mg/L (*p* = 0.0023).

Correlation analysis between all four hemogram ratios shows that NLR is correlated with the other three independently of mortality (NLR vs PLR, *ρ =* 0.7, *p* < 0.001; NLR vs NPR, *ρ =* 0.669, *p <* 0.001; NLR vs SII, *ρ =* 0.894, *p <* 0.001). However, PLR is correlated with SII (*ρ =* 0.816, *p <* 0.001) but not with NPR (*ρ =* 0.013, *p* = 0.53). Finally, NPR and SII showed a significant but weak correlation (*ρ =* 0.424, *p <* 0.001) (Fig. S4).

As expected, the hemogram-derived ratios were correlated with other hemogram parameters (Figs. S5A and B).

## Discussion

The health crisis caused by the COVID 19 pandemic has been unparalleled in our lifetime. Early identification of patients at risk of severe COVID-19 is essential to consider early aggressive interventions. We propose the use of simple hemogram analysis to obtain hemogram-derived ratios as well as their evolution to identify patients at risk of ICU admission. According the results obtained NPR is most useful hemogram-derived ratio to predict ICU admission.

Various laboratory parameters have been shown to identify high risk COVID-19 patients at risk of ICU admission and/or death. In our study patients admitted to ICU showed significantly higher values of LDH, CRP, APTT and D-Dimer. These results are consistent with previous studies [11, 20].

ICU patients also presented higher white blood cell count and neutrophils, while the inverse relationship was seen in non-neutrophil white blood cell series (low lymphocyte, monocyte and eosinophil ranges) and platelet levels (Table 2), similar results have been published previously [9, 16].

Many of these parameters reflect the patient’s inflammatory response to SARS-CoV-2 infection. A vast body of evidence shows that severe COVID-19 present an underlying hyperinflammatory response driving a cytokine release storm resulting in multiorgan failure and death [21]. This form of microvascular obstructive thromboinflammatory syndrome has been proposed as the pathophysiological mechanism underlying the hyperinflammatory response.

Our results show the use of four combined hemogram-derived ratios as predictors of unfavorable clinical evolution in a large number of COVID-19 infected patients. Specifically, NLR, NPR, SII and PLR may be used in combination as indicators of the inflammatory and immunological status.

NLR has been used as inflammatory marker in the context of COVID-19 [14, 15, 20, 22–24], and its prognostic value stands out among our findings [12, 13].

NPR emphasizes the importance of the relationship between immune response and homeostasis [12, 13]. We hypothesize that a damaged and activated endothelium would increase the permeability and release of cytokines that would in turn increase the chemotaxis of inflammatory cells and signal blood cells to favor the repair. In this context, platelets and neutrophils are activated by soluble agonists and adhesive proteins via their surface receptors playing a determining role in microvascular occlusion during thromboinflammatory disease [25]. NPR has already been shown to predict in-hospital mortality [12, 13]. However, the role of neutrophils in thrombosis is increasingly recognized and more is known about the immunomodulatory properties of platelets in such a way that they interact with each other during infection, inflammation and thrombosis by modulating the functions of each [26]**.**

On the other hand, the dynamic evolution of the immune response to SARS-CoV-2 infection could be crucial in the evolution of COVID-19 patients. Previous publications have shown the utility of velocity of change of hemogram-derived ratios to predict mortality in COVID-19 patients [12, 13]. According to our results, the velocity of change of four hemogram-derived ratios during first days of hospital admission would be signaling a greater inflammatory state in patients who will later require ICU admission as these parameters together with hemogram-derived ratios at hospital admission have shown their usefulness as prognostic markers of inflammation in patients who ultimately required admission to ICU. In this sense, the hemogram is a tool within the reach of all hospitals and doctors who do not have the technical and material means to carry out complex immunological studies, which often produce late results. The analysis of the hemogram-derived ratios would provide much more information than could be extracted a priori by evaluating the parameters in isolation. We now know that it is crucial to initiate early anti-inflammatory treatment when the patient deteriorates and the hemogram could be an indicator of that signal that could indicate which patients could potentially benefit from earlier anti-inflammatory therapy.

Dynamic changes during the evolution of the disease distort the assessment of every therapeutic intervention, especially in retrospective studies. An increase in the frequency of use of a drug due to clinical aggravation may not be distinguished from an effect of that drug. It is well known that the mechanisms of action of corticosteroids translate into several effects on the response of the immune system, classically producing lymphopenia, neutrophilia and also decreasing cytokine production. In theory, this mechanism may explain an increase in the hemogram-derived ratios. However, various studies regarding the prognostic value of NLR in inflammatory diseases (for example, in Bechet’s disease or Alcoholic hepatitis), have shown a reduction in the ratio in patients under corticosteroid treatment [27, 28]. In our study, patients admitted to ICU presented significant differences in the use of corticosteroids as a result of clinical worsening after symptom onset. While corticosteroid treatment may account for some effect on velocity of change, it doesn’t influence on hemogram-derived ratios on admission. This approach highlights the need for dynamic assessment of patients and may clarify the role of uncontrolled and nonrandomized drugs in COVID-19 studies.

Our study presented several limitations. The results of the stratified analysis (Figs. 1A, S1B, S1C and S1D) suggest that age is a significant factor that influences on ICU admission, affecting the rest of the variables. This fact could explain the loss of significance of the NPR in the most robust multivariate analysis and the association with mortality of age over 70 years with sex, hypertension, Sa O2 > 90, LDH > 677 U/L and CRP > 131 mg/L. During the study period, due to the pandemic situation in Spain, there was limited access to ICU beds and ventilators, which could have conditioned some results since patients could have been candidates for ICU based on age, comorbidities and survival chances. However, NPR is the only hemogram-derived ratio that maintains predictive capacity in the lower risk strata (< 70 years, male sex, Sa02 > 90, LDH > 677 U/L and CRP > 131 mg/L), which implies that their degree of independence with respect to these variables is greater (Fig. 1A). After reviewing our data, the same reason could explain the mortality rate of patients not admitted to the ICU. Although this number is small, it represents a percentage that may be relevant for the final analysis. This data may derive from patients with a high number of comorbidities or extreme age, a priori without ICU admission criteria. The presence in our study of other patients without ICU criteria but with a favorable evolution is not well established, however, this fact reproduces the usual clinical practice at the present time. Similarly, the use of drugs such as corticosteroids, antivirals and other drugs were variable, and did not always respond to the same criteria during the study period. The present study was conducted in the absence of different virus strains and further studies will assess whether the scores and prognostic factors described so far are useful with the new virus variants. Finally, this is a retrospective study and lacks a control group, which limits the systematic adoption in routine clinical practice. Further comprehensive studies are needed to determine how useful are these blood tests at which future prognostic scores demonstrate usefulness in guiding treatment decisions.

## Conclusions

Different hemogram-derived ratios and their dynamic assessment may be useful as prognostic indicators as they are able to predict risk of ICU admission in COVID-19 patients. Therefore, as the hemogram is a tool within the reach of all hospitals and doctors, we propose the use of the four hemogram-derived ratios for the assessment of the patient affected by COVID-19, especially the ty NPR that could be very useful as a marker in the prognosis of this disease since it includes inflammatory and thrombotic biomarkers, the main mechanisms involved in the development of severe manifestations of COVID-19.

## Supplementary Information

**Additional file 1: Supplementary Fig. S1B.** Interactions and stratified analyses for NLR (neutrophil-lymphocyte ratio) adjusted to model A (Table 5) and conducted for age (< 70 and > 70 years), sex, High blood pressure (HBP) , oxygen saturation (< 90 and > 90%) (SatO_2_), and lactate dehydrogenase (LDH) and C-reactive protein (CRP) both categorized through their respective median values. **Supplementary Fig. S1C.** Interactions and stratified analyses for SII (systemic immune-inflammation index) adjusted to model A (Table 5) and conducted for age (< 70 and > 70 years), sex, High blood pressure (HBP), oxygen saturation (< 90 and > 90%) (SatO_2)_, and lactate dehydrogenase (LDH) and C-reactive protein (CRP) both categorized through their respective median values. **Supplementary Fig. S1D.** Interactions and stratified analyses for PLR (platelet-lymphocyte ratio) adjusted to model A (Table 5) and conducted for age (< 70 and > 70 years), sex, High blood pressure (HBP), oxygen saturation (< 90 and > 90%) (SatO_2)_, and lactate dehydrogenase (LDH) and C-reactive protein (CRP) both categorized through their respective median values. **Supplementary Fig. S2.** Patients Flowchart. **Supplementary Fig. S3.** ROC curves for the different hemogram-derived ratios and their respective areas under the curves (AUC). **Supplementary Fig. S4.** Correlation analysis between the four hemogram-derived ratios. **Supplementary Fig. S5A.** Correlation analysis between NLR and NPR and those variables that were significantly associated with ICU entry. **Supplementary Fig. S5B.** Correlation analysis between PLR and SII and those variables that were significantly associated with ICU entry.

**Additional file 2.**

## Data Availability

The datasets used and/or analysed during the current study are available from the corresponding author on reasonable request.

## Abbreviations

ICU: Intensive care Unit
NLR: Neutrophil-to-lymphocyte ratio
PLR: Platelet-to-lymphocyte ratio
SII: The systemic inflammation Index
NPR: Neutrophil-platelet ratio
AST: Aspartate aminotransferase
ALT: Lactate aminotransferase
LDH: Lactate dehydrogenase
CRP: C-reactive protein
HTA: Arterial hypertension
SaO2: Oxygen saturation
