# Supplementary figures and images for: Hemogram-derived ratios as prognostic markers of ICU admission in COVID-19
Source: BMC Emerg Med. 2021 Jul 27;21:89. doi: 10.1186/s12873-021-00480-w (PMC8314257; doi:10.1186/s12873-021-00480-w)

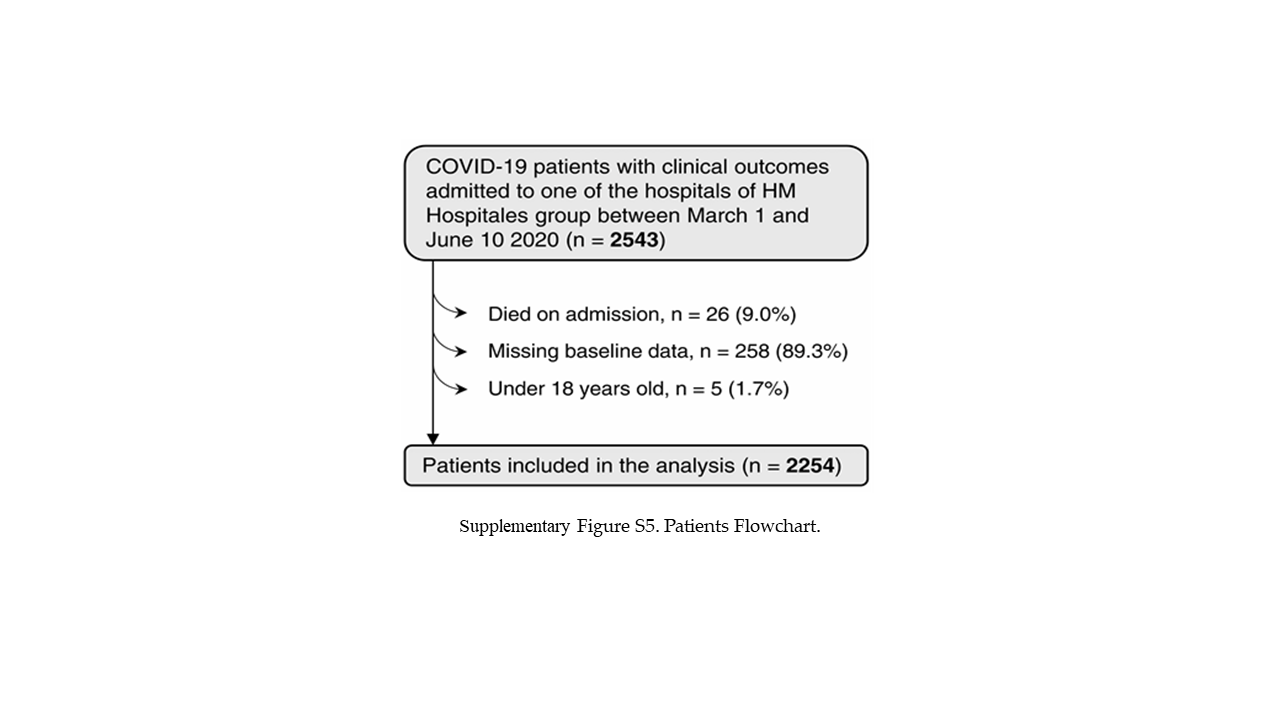

Supplement: Supplementary file 1 — Additional file 1: Supplementary Fig. S1B. Interactions and stratified analyses for NLR (neutrophil-lymphocyte ratio) adjusted to model A (Table 5) and conducted for age (< 70 and > 70 years), sex, High blood pressure (HBP) , oxygen saturation (< 90 and > 90%) (SatO2), and lactate dehydrogenase (LDH) and C-reactive protein (CRP) both categorized through their respective median values. Supplementary Fig. S1C. Interactions and stratified analyses for SII (systemic immune-inflammation index) adjusted to model A (Table 5) and conducted for age (< 70 and > 70 years), sex, High blood pressure (HBP), oxygen saturation (< 90 and > 90%) (SatO2), and lactate dehydrogenase (LDH) and C-reactive protein (CRP) both categorized through their respective median values. Supplementary Fig. S1D. Interactions and stratified analyses for PLR (platelet-lymphocyte ratio) adjusted to model A (Table 5) and conducted for age (< 70 and > 70 years), sex, High blood pressure (HBP), oxygen saturation (< 90 and > 90%) (SatO2), and lactate dehydrogenase (LDH) and C-reactive protein (CRP) both categorized through their respective median values. Supplementary Fig. S2. Patients Flowchart. Supplementary Fig. S3. ROC curves for the different hemogram-derived ratios and their respective areas under the curves (AUC). Supplementary Fig. S4. Correlation analysis between the four hemogram-derived ratios. Supplementary Fig. S5A. Correlation analysis between NLR and NPR and those variables that were significantly associated with ICU entry. Supplementary Fig. S5B. Correlation analysis between PLR and SII and those variables that were significantly associated with ICU entry. [file 12873_2021_480_MOESM1_ESM.zip › 12873_2021_480_MOESM1_ESM/Fig S2 (2).TIF]

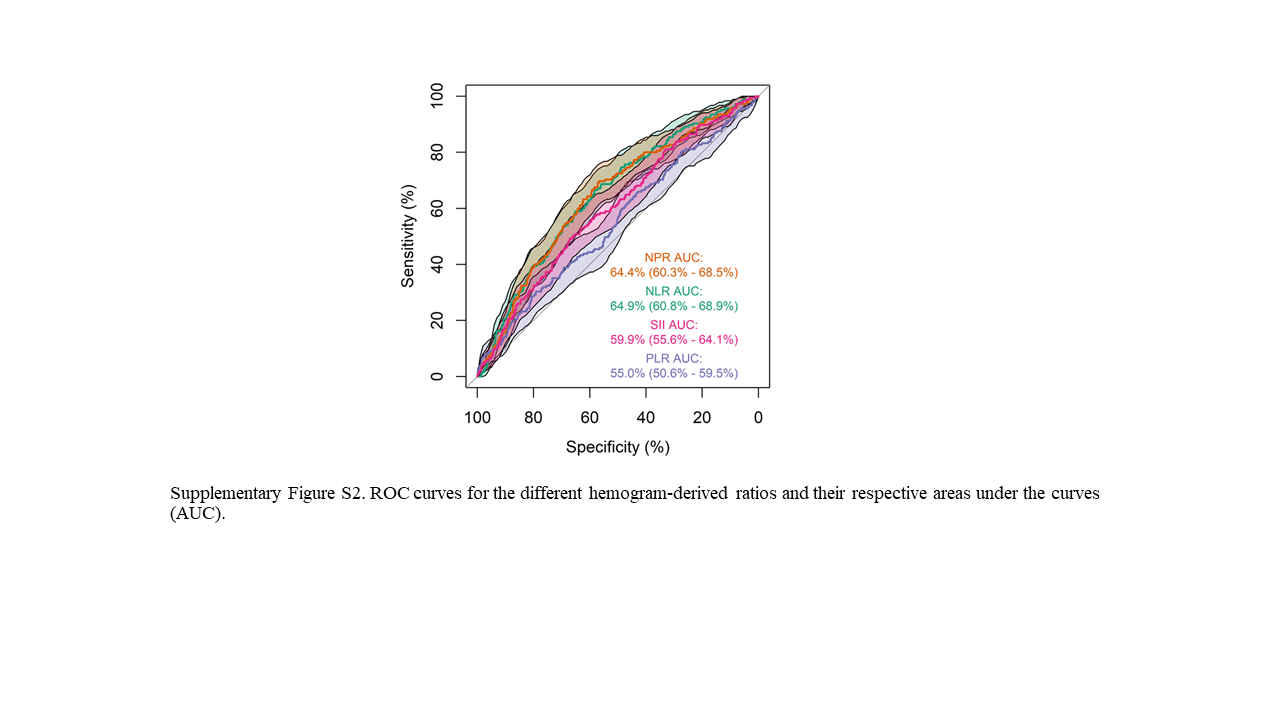

Supplement: Supplementary file 1 — Additional file 1: Supplementary Fig. S1B. Interactions and stratified analyses for NLR (neutrophil-lymphocyte ratio) adjusted to model A (Table 5) and conducted for age (< 70 and > 70 years), sex, High blood pressure (HBP) , oxygen saturation (< 90 and > 90%) (SatO2), and lactate dehydrogenase (LDH) and C-reactive protein (CRP) both categorized through their respective median values. Supplementary Fig. S1C. Interactions and stratified analyses for SII (systemic immune-inflammation index) adjusted to model A (Table 5) and conducted for age (< 70 and > 70 years), sex, High blood pressure (HBP), oxygen saturation (< 90 and > 90%) (SatO2), and lactate dehydrogenase (LDH) and C-reactive protein (CRP) both categorized through their respective median values. Supplementary Fig. S1D. Interactions and stratified analyses for PLR (platelet-lymphocyte ratio) adjusted to model A (Table 5) and conducted for age (< 70 and > 70 years), sex, High blood pressure (HBP), oxygen saturation (< 90 and > 90%) (SatO2), and lactate dehydrogenase (LDH) and C-reactive protein (CRP) both categorized through their respective median values. Supplementary Fig. S2. Patients Flowchart. Supplementary Fig. S3. ROC curves for the different hemogram-derived ratios and their respective areas under the curves (AUC). Supplementary Fig. S4. Correlation analysis between the four hemogram-derived ratios. Supplementary Fig. S5A. Correlation analysis between NLR and NPR and those variables that were significantly associated with ICU entry. Supplementary Fig. S5B. Correlation analysis between PLR and SII and those variables that were significantly associated with ICU entry. [file 12873_2021_480_MOESM1_ESM.zip › 12873_2021_480_MOESM1_ESM/Fig S3 (2).TIF]

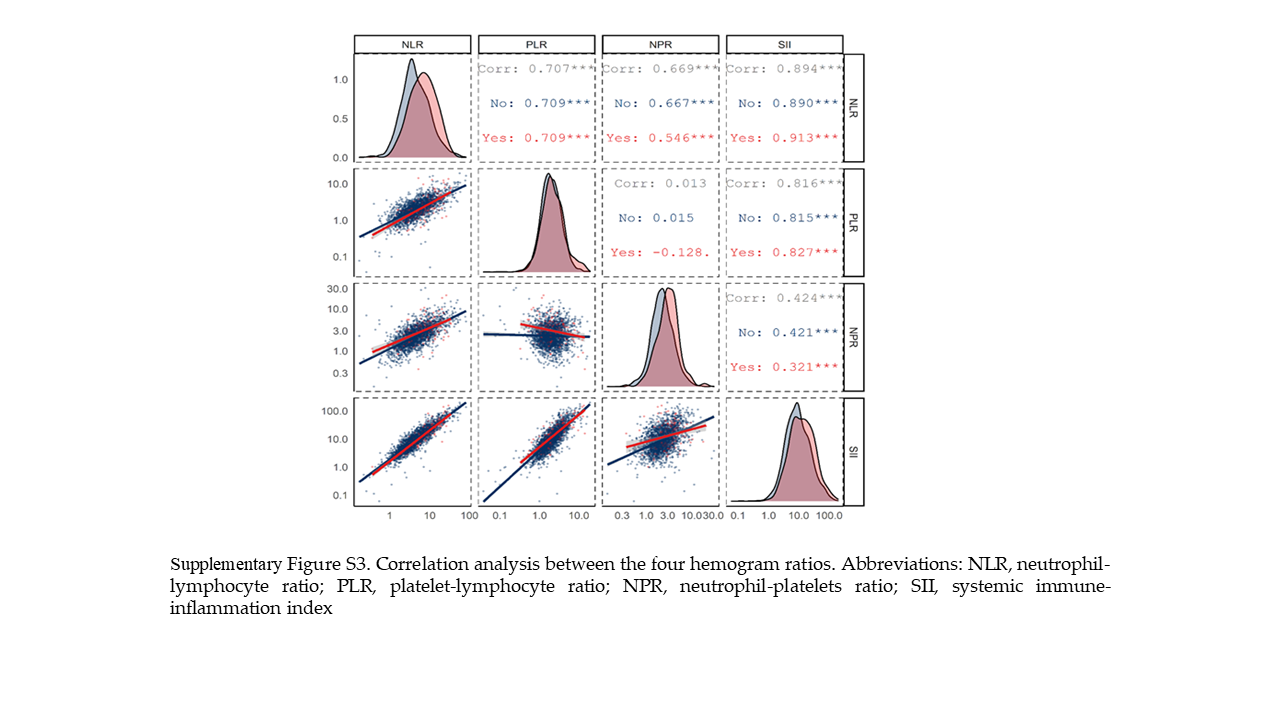

Supplement: Supplementary file 1 — Additional file 1: Supplementary Fig. S1B. Interactions and stratified analyses for NLR (neutrophil-lymphocyte ratio) adjusted to model A (Table 5) and conducted for age (< 70 and > 70 years), sex, High blood pressure (HBP) , oxygen saturation (< 90 and > 90%) (SatO2), and lactate dehydrogenase (LDH) and C-reactive protein (CRP) both categorized through their respective median values. Supplementary Fig. S1C. Interactions and stratified analyses for SII (systemic immune-inflammation index) adjusted to model A (Table 5) and conducted for age (< 70 and > 70 years), sex, High blood pressure (HBP), oxygen saturation (< 90 and > 90%) (SatO2), and lactate dehydrogenase (LDH) and C-reactive protein (CRP) both categorized through their respective median values. Supplementary Fig. S1D. Interactions and stratified analyses for PLR (platelet-lymphocyte ratio) adjusted to model A (Table 5) and conducted for age (< 70 and > 70 years), sex, High blood pressure (HBP), oxygen saturation (< 90 and > 90%) (SatO2), and lactate dehydrogenase (LDH) and C-reactive protein (CRP) both categorized through their respective median values. Supplementary Fig. S2. Patients Flowchart. Supplementary Fig. S3. ROC curves for the different hemogram-derived ratios and their respective areas under the curves (AUC). Supplementary Fig. S4. Correlation analysis between the four hemogram-derived ratios. Supplementary Fig. S5A. Correlation analysis between NLR and NPR and those variables that were significantly associated with ICU entry. Supplementary Fig. S5B. Correlation analysis between PLR and SII and those variables that were significantly associated with ICU entry. [file 12873_2021_480_MOESM1_ESM.zip › 12873_2021_480_MOESM1_ESM/Fig S4.tif]

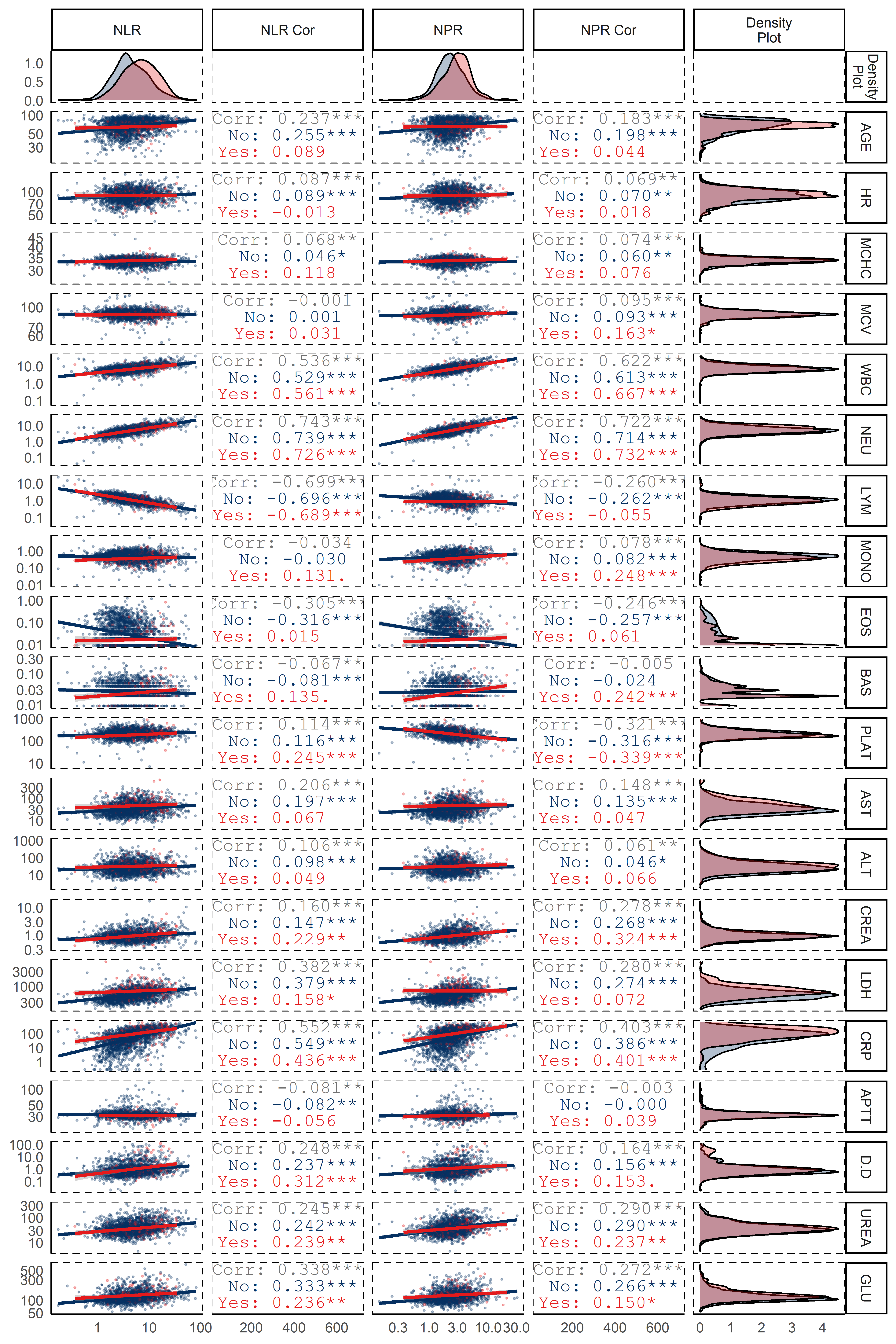

Supplement: Supplementary file 1 — Additional file 1: Supplementary Fig. S1B. Interactions and stratified analyses for NLR (neutrophil-lymphocyte ratio) adjusted to model A (Table 5) and conducted for age (< 70 and > 70 years), sex, High blood pressure (HBP) , oxygen saturation (< 90 and > 90%) (SatO2), and lactate dehydrogenase (LDH) and C-reactive protein (CRP) both categorized through their respective median values. Supplementary Fig. S1C. Interactions and stratified analyses for SII (systemic immune-inflammation index) adjusted to model A (Table 5) and conducted for age (< 70 and > 70 years), sex, High blood pressure (HBP), oxygen saturation (< 90 and > 90%) (SatO2), and lactate dehydrogenase (LDH) and C-reactive protein (CRP) both categorized through their respective median values. Supplementary Fig. S1D. Interactions and stratified analyses for PLR (platelet-lymphocyte ratio) adjusted to model A (Table 5) and conducted for age (< 70 and > 70 years), sex, High blood pressure (HBP), oxygen saturation (< 90 and > 90%) (SatO2), and lactate dehydrogenase (LDH) and C-reactive protein (CRP) both categorized through their respective median values. Supplementary Fig. S2. Patients Flowchart. Supplementary Fig. S3. ROC curves for the different hemogram-derived ratios and their respective areas under the curves (AUC). Supplementary Fig. S4. Correlation analysis between the four hemogram-derived ratios. Supplementary Fig. S5A. Correlation analysis between NLR and NPR and those variables that were significantly associated with ICU entry. Supplementary Fig. S5B. Correlation analysis between PLR and SII and those variables that were significantly associated with ICU entry. [file 12873_2021_480_MOESM1_ESM.zip › 12873_2021_480_MOESM1_ESM/FigS5A (1).png]

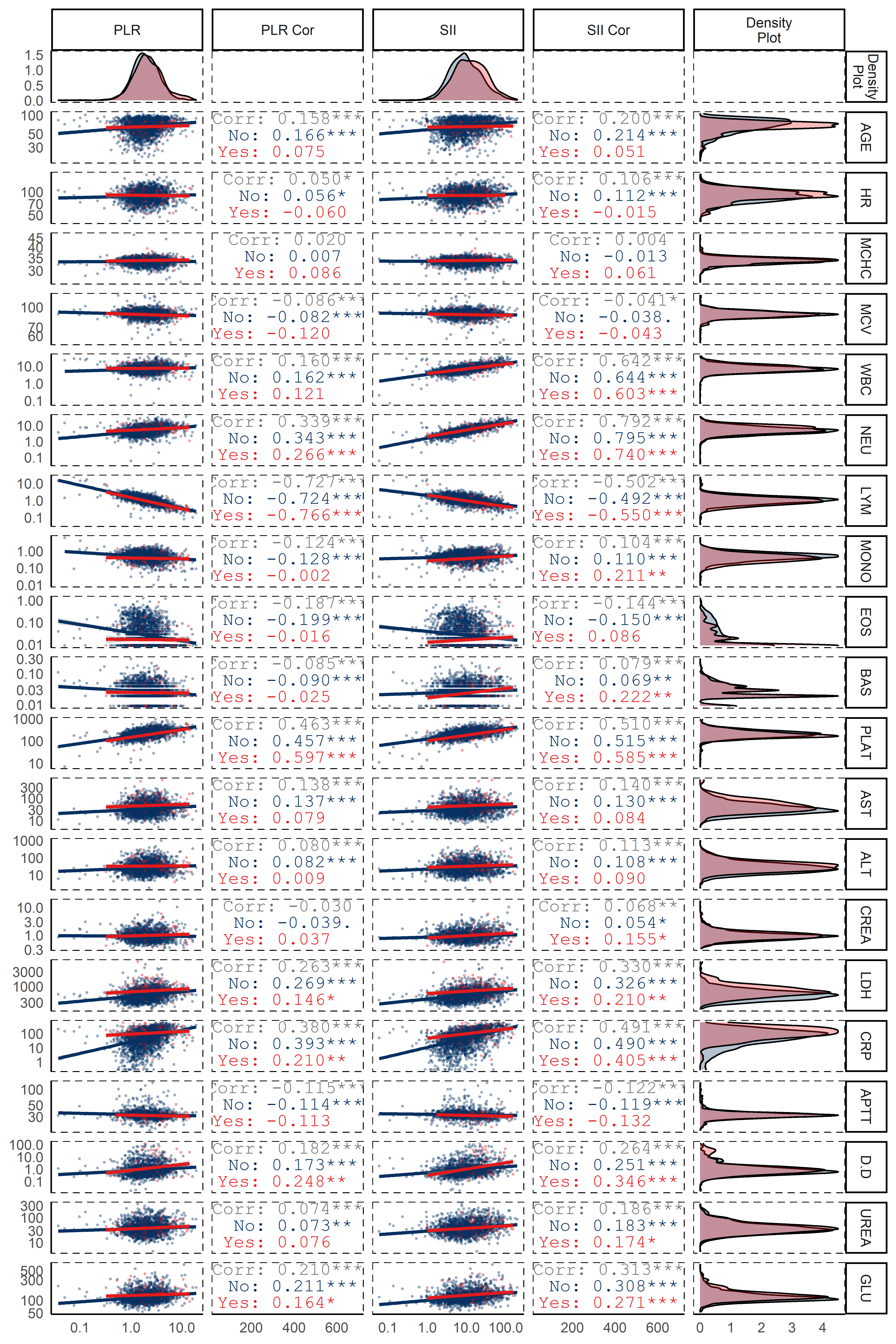

Supplement: Supplementary file 1 — Additional file 1: Supplementary Fig. S1B. Interactions and stratified analyses for NLR (neutrophil-lymphocyte ratio) adjusted to model A (Table 5) and conducted for age (< 70 and > 70 years), sex, High blood pressure (HBP) , oxygen saturation (< 90 and > 90%) (SatO2), and lactate dehydrogenase (LDH) and C-reactive protein (CRP) both categorized through their respective median values. Supplementary Fig. S1C. Interactions and stratified analyses for SII (systemic immune-inflammation index) adjusted to model A (Table 5) and conducted for age (< 70 and > 70 years), sex, High blood pressure (HBP), oxygen saturation (< 90 and > 90%) (SatO2), and lactate dehydrogenase (LDH) and C-reactive protein (CRP) both categorized through their respective median values. Supplementary Fig. S1D. Interactions and stratified analyses for PLR (platelet-lymphocyte ratio) adjusted to model A (Table 5) and conducted for age (< 70 and > 70 years), sex, High blood pressure (HBP), oxygen saturation (< 90 and > 90%) (SatO2), and lactate dehydrogenase (LDH) and C-reactive protein (CRP) both categorized through their respective median values. Supplementary Fig. S2. Patients Flowchart. Supplementary Fig. S3. ROC curves for the different hemogram-derived ratios and their respective areas under the curves (AUC). Supplementary Fig. S4. Correlation analysis between the four hemogram-derived ratios. Supplementary Fig. S5A. Correlation analysis between NLR and NPR and those variables that were significantly associated with ICU entry. Supplementary Fig. S5B. Correlation analysis between PLR and SII and those variables that were significantly associated with ICU entry. [file 12873_2021_480_MOESM1_ESM.zip › 12873_2021_480_MOESM1_ESM/FigS5B (1).png]

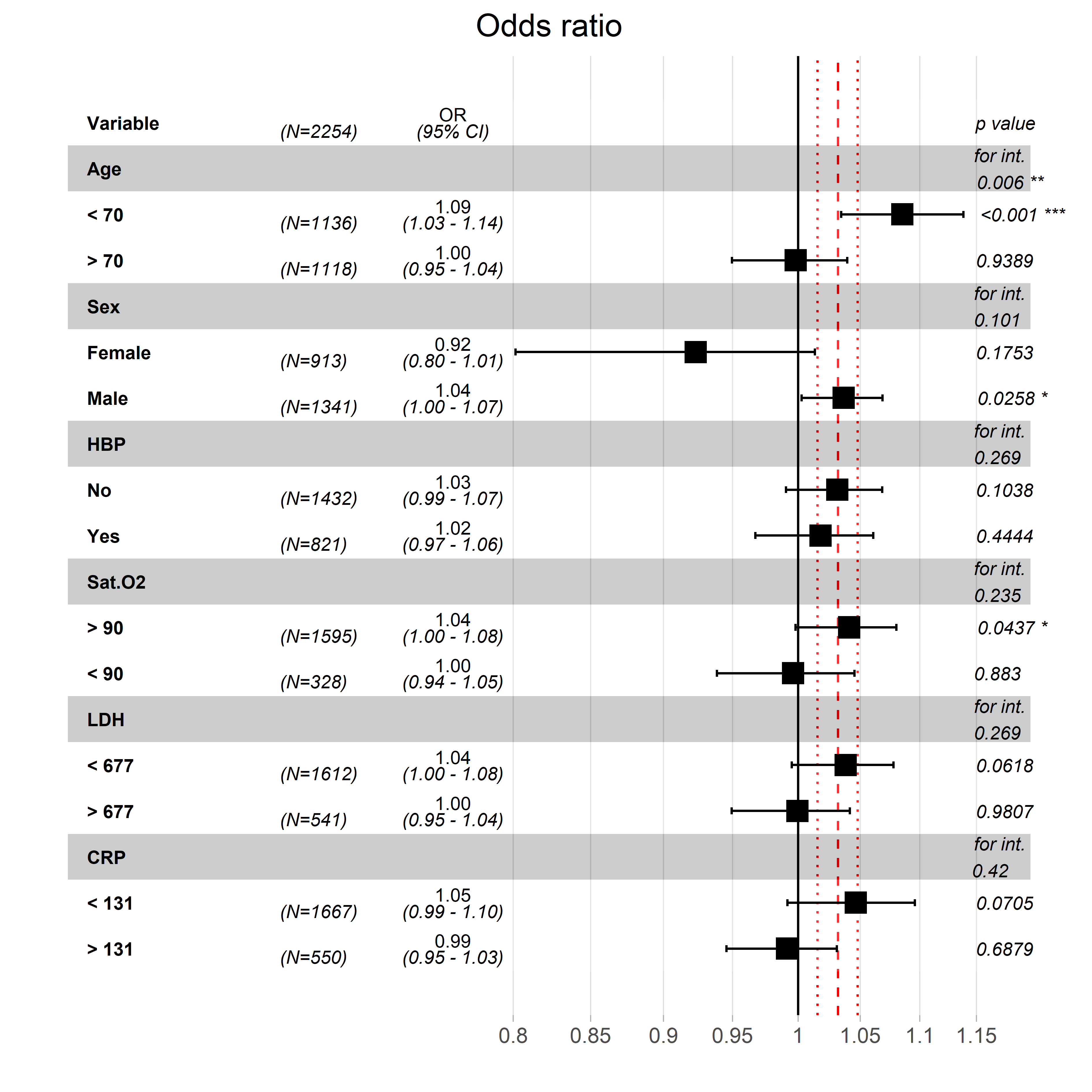

Supplement: Supplementary file 1 — Additional file 1: Supplementary Fig. S1B. Interactions and stratified analyses for NLR (neutrophil-lymphocyte ratio) adjusted to model A (Table 5) and conducted for age (< 70 and > 70 years), sex, High blood pressure (HBP) , oxygen saturation (< 90 and > 90%) (SatO2), and lactate dehydrogenase (LDH) and C-reactive protein (CRP) both categorized through their respective median values. Supplementary Fig. S1C. Interactions and stratified analyses for SII (systemic immune-inflammation index) adjusted to model A (Table 5) and conducted for age (< 70 and > 70 years), sex, High blood pressure (HBP), oxygen saturation (< 90 and > 90%) (SatO2), and lactate dehydrogenase (LDH) and C-reactive protein (CRP) both categorized through their respective median values. Supplementary Fig. S1D. Interactions and stratified analyses for PLR (platelet-lymphocyte ratio) adjusted to model A (Table 5) and conducted for age (< 70 and > 70 years), sex, High blood pressure (HBP), oxygen saturation (< 90 and > 90%) (SatO2), and lactate dehydrogenase (LDH) and C-reactive protein (CRP) both categorized through their respective median values. Supplementary Fig. S2. Patients Flowchart. Supplementary Fig. S3. ROC curves for the different hemogram-derived ratios and their respective areas under the curves (AUC). Supplementary Fig. S4. Correlation analysis between the four hemogram-derived ratios. Supplementary Fig. S5A. Correlation analysis between NLR and NPR and those variables that were significantly associated with ICU entry. Supplementary Fig. S5B. Correlation analysis between PLR and SII and those variables that were significantly associated with ICU entry. [file 12873_2021_480_MOESM1_ESM.zip › 12873_2021_480_MOESM1_ESM/Figure S1B.png]

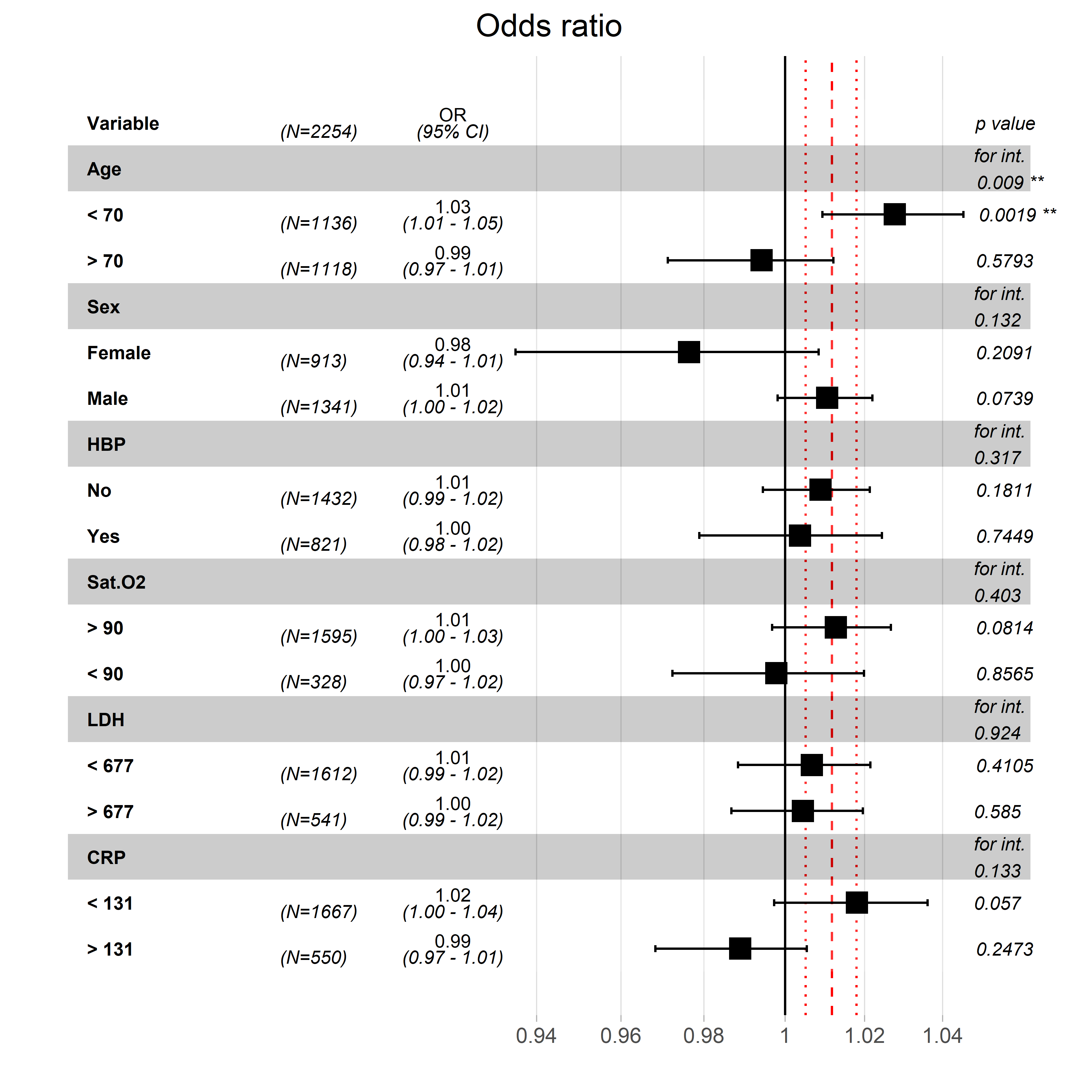

Supplement: Supplementary file 1 — Additional file 1: Supplementary Fig. S1B. Interactions and stratified analyses for NLR (neutrophil-lymphocyte ratio) adjusted to model A (Table 5) and conducted for age (< 70 and > 70 years), sex, High blood pressure (HBP) , oxygen saturation (< 90 and > 90%) (SatO2), and lactate dehydrogenase (LDH) and C-reactive protein (CRP) both categorized through their respective median values. Supplementary Fig. S1C. Interactions and stratified analyses for SII (systemic immune-inflammation index) adjusted to model A (Table 5) and conducted for age (< 70 and > 70 years), sex, High blood pressure (HBP), oxygen saturation (< 90 and > 90%) (SatO2), and lactate dehydrogenase (LDH) and C-reactive protein (CRP) both categorized through their respective median values. Supplementary Fig. S1D. Interactions and stratified analyses for PLR (platelet-lymphocyte ratio) adjusted to model A (Table 5) and conducted for age (< 70 and > 70 years), sex, High blood pressure (HBP), oxygen saturation (< 90 and > 90%) (SatO2), and lactate dehydrogenase (LDH) and C-reactive protein (CRP) both categorized through their respective median values. Supplementary Fig. S2. Patients Flowchart. Supplementary Fig. S3. ROC curves for the different hemogram-derived ratios and their respective areas under the curves (AUC). Supplementary Fig. S4. Correlation analysis between the four hemogram-derived ratios. Supplementary Fig. S5A. Correlation analysis between NLR and NPR and those variables that were significantly associated with ICU entry. Supplementary Fig. S5B. Correlation analysis between PLR and SII and those variables that were significantly associated with ICU entry. [file 12873_2021_480_MOESM1_ESM.zip › 12873_2021_480_MOESM1_ESM/Figure S1C.png]

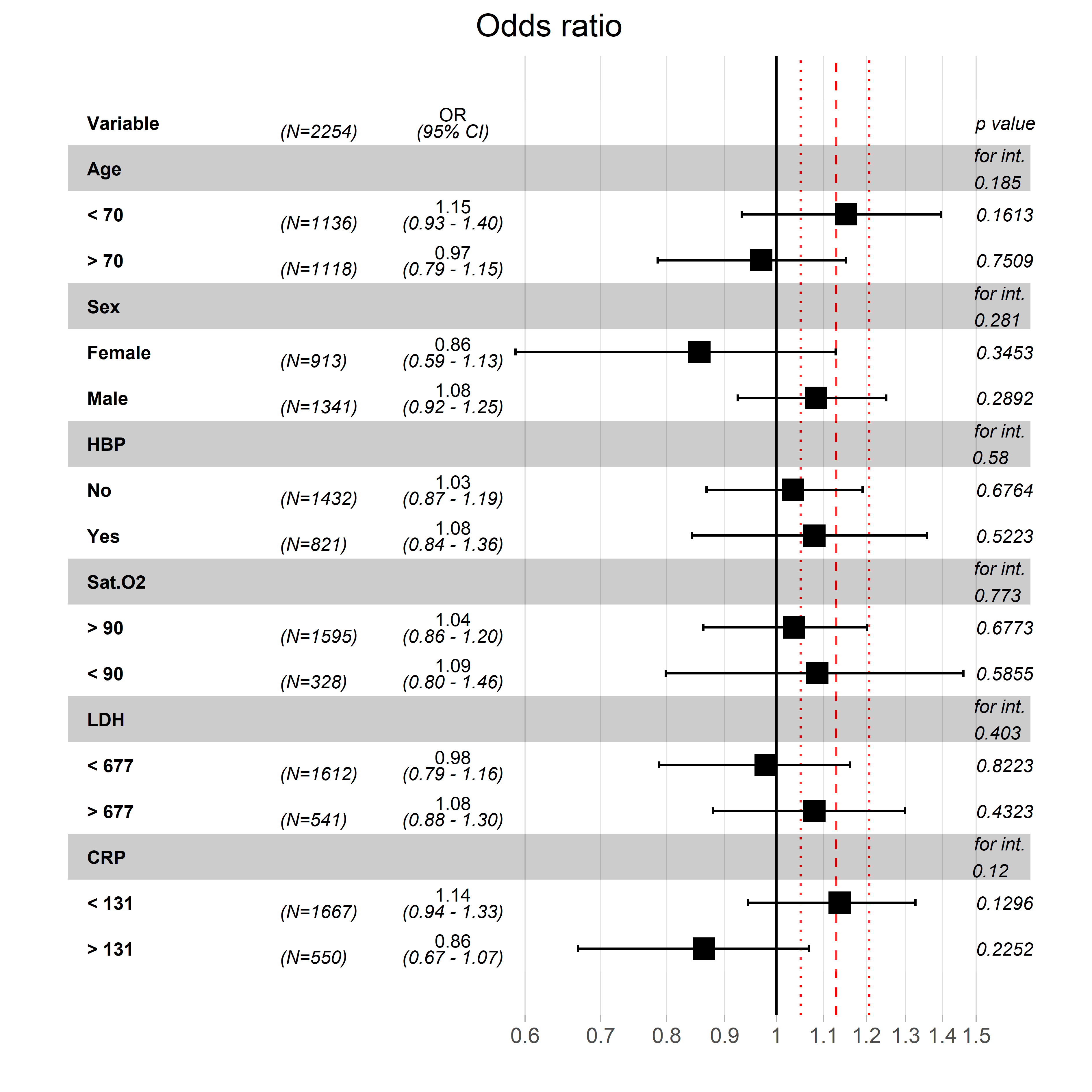

Supplement: Supplementary file 1 — Additional file 1: Supplementary Fig. S1B. Interactions and stratified analyses for NLR (neutrophil-lymphocyte ratio) adjusted to model A (Table 5) and conducted for age (< 70 and > 70 years), sex, High blood pressure (HBP) , oxygen saturation (< 90 and > 90%) (SatO2), and lactate dehydrogenase (LDH) and C-reactive protein (CRP) both categorized through their respective median values. Supplementary Fig. S1C. Interactions and stratified analyses for SII (systemic immune-inflammation index) adjusted to model A (Table 5) and conducted for age (< 70 and > 70 years), sex, High blood pressure (HBP), oxygen saturation (< 90 and > 90%) (SatO2), and lactate dehydrogenase (LDH) and C-reactive protein (CRP) both categorized through their respective median values. Supplementary Fig. S1D. Interactions and stratified analyses for PLR (platelet-lymphocyte ratio) adjusted to model A (Table 5) and conducted for age (< 70 and > 70 years), sex, High blood pressure (HBP), oxygen saturation (< 90 and > 90%) (SatO2), and lactate dehydrogenase (LDH) and C-reactive protein (CRP) both categorized through their respective median values. Supplementary Fig. S2. Patients Flowchart. Supplementary Fig. S3. ROC curves for the different hemogram-derived ratios and their respective areas under the curves (AUC). Supplementary Fig. S4. Correlation analysis between the four hemogram-derived ratios. Supplementary Fig. S5A. Correlation analysis between NLR and NPR and those variables that were significantly associated with ICU entry. Supplementary Fig. S5B. Correlation analysis between PLR and SII and those variables that were significantly associated with ICU entry. [file 12873_2021_480_MOESM1_ESM.zip › 12873_2021_480_MOESM1_ESM/Figure S1D.png]
