# Supplementary material for: Hemogram-derived ratios as prognostic markers of ICU admission in COVID-19
Source: BMC Emerg Med. 2021 Jul 27;21:89. doi: 10.1186/s12873-021-00480-w (PMC8314257; doi:10.1186/s12873-021-00480-w)
Supplement: Supplementary file 2 — Additional file 2. [file 12873_2021_480_MOESM2_ESM.docx]

Appendix 1.

*Online survey*

| Outcome | Questions | | Answers |
| --- | --- | --- | --- |
| Demographic information | 1 | What is your actual position? (*FR: Quel est votre poste actuel ?)* | nurse assistant, nurse, psychiatric nurse, head nurse, social worker, head social worker, intern physician, resident physician, head physician, psychiatrist, psychologist, other |
|  | 2 | In which ED are you working? (*F : Dans quel département d’urgence travaillez-vous ?*) | ED with CM/ ED without CM |
|  | 3 | How old are you? | List |
|  | 4 | Gender | Male/ female |
|  | 5 | How many years have you been working in the ED? | Number of years |
|  | 6 | What type of patients does your ED accept? (*FR: Quelle catégorie de patients accueillez-vous dans le service des urgences dans lequel vous travaillez?)* | acute emergencies ambulatory emergencies  psychiatric emergencies  Other: text |
| Extent FUED are perceived as an issue | 7 | To what extent are FUED are problematic in your ED? (*FR: Dans quelle mesure les patients HCSU posent-ils un problème dans votre service ?)* | An extremely important matter  An important matter  A rather important matter  A matter not important |

| Appendix 1.  *Online survey* | | | |
| --- | --- | --- | --- |
| Outcome |  | Question | Answers |
| Perceived FUED-related work difficulties | 8 | FUED might contribute to several work difficulties. Over the past 12 months, to what extent have you been faced the following difficulties relating to FUED? (*FR: Les HCSU peuvent être à l'origine de diverses difficultés sur le terrain. Au cours des 12 derniers mois, dans quelle mesure avez-vous été confronté aux difficultés suivantes ? (Uniquement en lien avec les HCSU)*)  8.1 A feeling of helplessness related to FUED (*FR : Un sentiment d’impuissance face à ces patients*)  8.2 A feeling of failure facing FUED (*FR: Un sentiment de mise en échec*)  8.3 A feeling of ED staff ineffectiveness towards FUED issue (*FR: Un sentiment d'inefficacité des équipes soignantes*)  8.4 Exhaustion of ED staff in relation to FUED (*FR: L'épuisement des équipes soignantes*)  8.5 Increased ED overcrowding due to FUED (*FR: L'augmentation de la surcharge des urgences dû aux HCSU*)  8.6 Difficulties in creating a therapeutic alliance with FUED (*FR: Des difficultés à créer une alliance thérapeutique*)  8.7 Difficulties related to FUEDs’ social situations (*FR: Des difficultés émergentes de leur situation sociale*)  8.8 Difficulties related to FUEDs’ psychiatric comorbidities *(FR: Des difficultés émergentes de leurs comorbidités psychologiques/psychiatriques*)  8.9 Difficulties related to FUEDs’ substance use (e.g., alcohol, drugs) (*FR: Des difficultés émergentes de leurs addictions (p.ex. alcool, drogue)*)  8.10 A health condition complicated by FUEDs’ negative behavior (e.g., aggressiveness, impatience) (*FR: Une prise en charge compliqué par leur attitude négative (p.ex. hétéro-agressivité, impatience, ...)*)  8.11 An inefficient medical care for FUED (*FR: Une prise en charge inefficace aux urgences*)  8.12 A lack of resolution in the ED regarding a FUED (*FR: Un manque de solution aux urgences pour répondre aux besoins de ces patients*)  8.13 A risk of banalization of a FUEDs’ health condition (*FR: La création d'un risque de banalisation de l'état de santé de ces patients*)  8.14 Difficulties to redirect FUEDs (*FR: Difficultés à orienter les patients à leur sortie des urgences*)  8.15 Difficulties to collaborate with FUEDs’ healthcare network (*FR: Difficultés à collaborer avec le réseau de ces patients)* | Likert-type scale, 0 = *strongly disapprove* to *10 = strongly approve* |
| Appendix 1.  *Online survey* | | | |
| Outcome |  | Questions | Answers |
| Perceived legitimacy of FUED ED visits FUED | 9 | To what extent do you consider the FUEDs you have encountered require an ED consultation ? (*FR: Dans quelle mesure, selon vous, les HCSU présentent-ils des problèmes nécessitant la consultation de services d’urgence?*) | Likert-type scale, 0 = *strongly disagree*  to 10 = *strongly agree* |
| Level of perceived knowledge | 10 | How do you rate your level of knowledge regarding FUED issue? (*FR: Comment évalueriez-vous votre niveau de connaissance de la problématique des HCSU?)* | Likert-type scale, 0 = *not at all aware* to *4 = extremely aware* |
| Actual knowledge score | 11 | In your opinion, what are FUED characteristics? (*FR: Selon vous, quelles sont les caractéristiques des patients HCSU?*)  11.1 Use ED in an inappropriate way (*FR: Utilisent les urgences de façon inappropriée*)  11.2 Present a high social and medical vulnerability (*FR: Présentent une vulnérabilité sociale et médicale élevée*)  11.3 Frequently suffer from chronic illnesses (*FR: Souffrent souvent d'une maladie chronique*)  11.4 Have a higher mortality rate compared to other users of the ED (*FR: Ont un taux de mortalité plus élevé comparé aux autres usagers*)  11.5 Frequently have no general practitioner (*FR: N'ont souvent pas de médecin traitant*)  11.6 Frequently perceive they are discriminated against (*FR: Perçoivent souvent des discriminations à leur égard*)  11.7 Come from disadvantaged socioeconomic classes (*FR: Sont souvent issus de couches socio-économiques défavorisées*)  11.8 Frequently present with psychiatric conditions (*FR: Présentent plus souvent des pathologies psychiatriques*)  11.9 Frequently live close to ED (< 10 km) (*FR: Habitent souvent à côté des urgences (<10km)*)  11.10 Are heavy customers of healthcare system (*FR: Sont des gros consommateurs du système de santé*)  11.11 Don’t have a health insurance (*FR: Ne Bénéficient pas d'une assurance maladie*)  11.12 Frequently present with substance use disorders (*FR: Présentent plus souvent des addictions*)  11.13 Are more complexe patients compared to other users of the ED (*FR: Sont des patients plus difficiles que les autres*)  11.14 Have no job (*FR: N'ont pas un emploi*) | Likert-type scale, 0 = *strongly disagree*  to 10 = *strongly agree* |

Appendix 2.

*Qualitative quotes*

| Participants | Original quotes | Translated quotes |
| --- | --- | --- |
| Physician 3, no-CM | « Ce n’est pas une population sur laquelle on est informé. J’ai peu entendu parler des patients récurrents comme une entité ou une spécificité pour un patient qui mènerait à une spécificité de prise en charge » | “It is not a population we are informed about. I have heard very little of recurrent (ED) patients as a specific entity or a type of patient that would require specific management.” |
| Nurses’ focus group, CM | « J’aimerais juste revenir sur : Un patient récurrent c’est quoi la définition finalement, combien de fois ? Parce qu’on peut avoir des patients récurrents (…) que l’on ne va pas forcément signaler. » | “I would like to come back: a recurring (ED) patient, what is the definition in the end, how many times? Because we have recurring patients (…) that we will not necessarily report”. |
| Physician 3, no-CM | « Une problématique importante en termes de nombre de patients et de fréquence de visites aux urgences » | “It is an important problem in terms of the number of patients and frequency of emergency room visits” |
| Physician 3, no-CM | « On n’a effectivement pas de possibilité par rapport à cette problématique. Ça demande une certaine prise en charge qui n’est pas la même que les autres patients » | “We effectively have no possibilities in relation to this problem. It requires a certain level of care that is not the same as other patients.” |
| Physician 1, CM | « La place de ces gens ce n’est pas les urgences » | “The place for these people is not the emergency room” |
| Physician 2, no-CM | « C’est des gens qui sont finalement en bonne santé, qui n’ont pas de grosses comorbidités somatiques.» | “It’s people who are in good health (…) don’t have many somatic comorbidities.” |
| Physician 1, no-CM | « Je pense qu’il y a une inquiétude de la part du patient, et il faut y répondre » | “I think there is a concern from the patient, and this must be addressed” |
| Physician 3, CM | « La première chose chez ces patients à par récurrent c’est : chronophage, agacent et générateur de contre-transferts négatifs. » | “The first thing in these patients with recurrences is: time consuming, annoying and generates negative countertransference.” |
| Physician 2,  CM | « (…) On n’arrive pas à les guérir tout simplement. Donc ça oui, ça éveille un sentiment d’impuissance dans l’équipe et de fatigue » | “(…) We just can’t heal them. So yes, it awakens a feeling of helplessness in the team and fatigue” |
| Nurses’ focus group, CM | « Du côté du case management, je trouve que ce n’est pas ça, moi je n’étais pas au courant qu’ils faisaient tout cela en fait (…)on a moins d’information sur vraiment ce que « les populations vulnérables » font concrètement (…). On est potentiellement biaisé parce que c’est tout à coup des patients qu’on ne voit plus et on ne réalise pas forcément  » | “The case management side, I don't think it is yet, I wasn't aware that they were actually doing all this (…) we have less information on what the “vulnerable populations” team (i.e., CM team) do (…) We're potentially biased because it's suddenly patients we don't see anymore and we don't necessarily realize” |
| Nurses’ focus group, CM | « Ça ne va pas vous êtes angoissé, Temesta® et puis vous rentrez chez vous, (…) quand vous voyez quelqu’un qui vient de manière récurrente (…) et un quart d’heure après redescend de la psychiatrie de liaison, on ne peut pas dire que ce soit une prise en charge efficiente ou bien faite » | “It's not going okay, you're anxious, Temesta® and then you go home, (...) when you see someone who comes in a recurring way (...) and a quarter of an hour after coming down from psychiatric consultation, you can't say it's efficient or well-done care” |
